# Variability of Accessory Pathway Refractory Periods: What Should be the Criteria for Ablation in Asymptomatic WPW

**DOI:** 10.1016/s0972-6292(16)30499-5

**Published:** 2012-05-20

**Authors:** Ajit Kumar Valaparambil

**Affiliations:** Professor of Cardiology, Sree Chitra Tirunal Institute for Medical Sciences and Technology, Trivandrum, Kerala

**Keywords:** Refractory Periods, Accessory Pathway, Criteria for Ablation

The rationale behind active treatment and ablation in asymptomatic WPW is to prevent sudden cardiac death (SCD). SCD in asymptomatic WPW syndrome has been noted to vary from 0.15 to 0.39% over 3-10 years follow up [1,2].

SCD happens in WPW in relation to rapid pre-excited atrial fibrillation (AF) with fast ventricular response. It happens in 50% of SCD as the first manifestation. Hence, though the incidence of SCD is low in WPW, the same is avoidable in suitably selected cases. It can presently be taken care of, by radiofrequency (RF) ablation of the bypass tract without significant morbidity.

What then are the criteria to select patients of Asymptomatic WPW who may be predisposed to SCD? The markers identified for SCD include: 1. Anterograde effective refractory period of accessory pathway (APERP) 240- 250ms; (on isoproternol APERP <200ms?) 2. Shortest Pre-excited RR interval (SPERRI) less than 250ms during or induced AF. 3. Multiple accessory pathways 4. Ebsteins anomaly 5. Familial WPW syndrome. The Non invasive parameters of a presumed long refractory period of bypass tract (>250ms) include: 1. Intermittent pre-excitation. 2. Disappearance of pre-excitation during exercise and 3. Disappearance of pre-excitation with procainamide. The predictive value of the non invasive parameters is low and when combined with the low risk of RF ablation, most electrophysiologists rely on invasive testing to risk stratify asymptomatic patients. In a European survey 44% of EP centers perform invasive approach as the first line approach [3] in asymptomatic WPW.

Regardless of how the EP protocol is set, one of the specific parameter taken is anterograde ERP <240ms at baseline to decide RF ablation. Additional parameters include minimum RR interval in AF: SPERRI <250ms, and AF induction. Considering all boundary parameters, the best binary discriminators are APERP <240ms at baseline and SPERRI <250ms, which we follow at our centre. In perhaps the best prospective follow up study in children age 8-12 yrs, with a follow up of 57 months, Pappone et al [4-7] identified the following 1. Tachyarrhythmia inducibility. 2. APERP <240ms 3. Multiple accessory pathways; as independent risk factors for life threatening events. Of these APERP and multiple bypass tracts were independent predictors by multivariate analysis.

The "PACES /HRS Consensus statement on the management of the Asymptomatic Young Patient with a WPW Electrocardiographic pattern" published this week [9] has noted that SPERRI and APERP remain important baseline parameters in risk stratifying asymptomatic WPW patients, with high sensitivity and negative predictive value; but with low specificity and positive predictive value. They have observed that SPERRI <250ms is superior to APERP <240ms in discriminating patients at risk for SCD or VF. The consensus statement has recommended that young patients with SPERRI <250ms in AF are at increased risk for SCD and RF Catheter ablation is a reasonable option ( Class IIA, Level of evidence B/C ).

Therefore the most important binary parameter to the invasive electrophysiologist is SPERRI <250ms and APERP <240ms at baseline (without isoproternol) to decide RF Ablation.

The important question raised in this issue by Oliver C et al in this issue of the journal [8] is the stability of this parameter of APERP. Does it change temporally at two different occasions? Does the APERP assessed by isoproternol also change with time? In short: is the parameter of baseline APERP stable and to put it clinically, can it be accepted as a value to decide RF ablation or otherwise in asymptomatic WPW.

The changes of baseline APERP temporally in the basal state, as noted by the authors, [8] are variable, but not significant enough, to warrant different approaches or multiple studies to decide the need for RF ablation. A minor group of patients (15% had a APERP <240ms at the second study) will be left out with an ambiguous decision for medical follow up if the first baseline APERP is chosen. But it is also likely that this group will be picked for further evaluation and not necessarily present as SCD.

The response to APERP on isoproternol has been studied by multiple authors; though acceptance of a standard value has not been universally agreed upon. A value of APERP <200ms on isoproternol has been noted as a cut off value, but the dose of the drug has ranged from 1-20μg per minute. The authors [8] have noted significant variations in the APERP values temporally after isoproternol infusion. A significant group (with APERP >200ms) at first study- 33% had APERP <200ms at the second study. The conclusion would necessarily imply that the ERP values on isoproternol cannot be relied for assessing risk in the asymptomatic WPW. The variations are likely to be related to the status of the autonomic system that will modulate the electrophysiological properties of the bypass tract and the AV Node.

I would like to conclude by noting that APERP estimations are temporally variable. The variations are more pronounced during isoproternol infusion. As a matter of fact all EP parameters ranging from venricular ERP to VT Inducibility are temporally variable; what is important is to know the significance of a baseline parameter in relation to the natural history of the disease. On the background of the natural history studies by previous authors, the variations in the basal state are acceptable and a value of APERP <240ms can serve as a binary cut off value to indicate and select a potentially high risk group that can undergo RF ablation to reduce SCD. EP studies of APERP on isoproternol show significant variations temporally and cannot be relied upon as a sole predictor of future risk, independently; however it can contribute to the basal study. . Based on the Consensus Statement and Recommendations SPERRI <250 ms can serve as a parameter to select patients for catheter ablation [9] and is superior to APERP <240ms at baseline. Other risk factors too, as noted above, may be taken into consideration in the decision making process. The basal APERP study, inspite of its limitations, can guide the decision to ablate or not in Asymptomatic WPW.
